# Seroprevalence of Specific SARS-CoV-2 Antibodies during Omicron BA.5 Wave, Portugal, April–June 2022

**DOI:** 10.3201/eid2903.221546

**Published:** 2023-03

**Authors:** Irina Kislaya, Aryse Melo, Marta Barreto, Camila Henriques, Carlos Aniceto, Carla Manita, Sara Ramalhete, João Almeida Santos, Sofia Soeiro, Ana Paula Rodrigues, ISN4COVID-19 Group

**Affiliations:** Instituto Nacional de Saúde Doutor Ricardo Jorge, Lisbon, Portugal

**Keywords:** COVID-19, 2019 novel coronavirus disease, coronavirus disease, severe acute respiratory syndrome coronavirus 2, SARS-CoV-2, viruses, respiratory infections, zoonoses, seroepidemiological survey, IgG, seroprevalence, Portugal, Omicron, BA.5

## Abstract

After the rapid spread of SARS-CoV-2 BA.5 Omicron lineage in Portugal, we developed a seroepidemiologic survey based on a sample of 3,825 residents. Results indicated that from April 27 through June 8, 2022, the estimated seroprevalence of SARS-CoV-2 nucleocapsid or spike IgG was 95.8%, which indicates a high level of protection.

Serial seroepidemiologic surveys contribute information about pandemic dynamics; monitoring population-level SARS-CoV-2 antibody distribution establishes trends in postinfection and vaccine-induced immunity. Such surveys are essential to integrated surveillance systems for respiratory infections (*1*).

During May 2020–June 2022, the National Health Institute Doutor Ricardo Jorge, in partnership with the National Clinical Pathology Laboratories Association, the Portuguese Association of Clinical Analysts, and a nationwide network of public hospitals, conducted 4 serial seroepidemiologic surveys (ISN1COVID-19, ISN2COVID-19, ISN3COVID-19, and ISN4COVID-19). Number of study participants ranged from 2,301 to 8,463 residents of Portugal (*2*–*4*) (Figure 1). The fourth survey (ISN4COVID-19) was conducted from April 27, 2022, through June 8, 2022, after the mandatory mask mandate was lifted and during rapid spread of the SARS-CoV-2 BA.5 Omicron lineage (*5*) (Appendix Figure 1) and the ongoing second booster vaccination campaign (*6*) (Appendix Figure 2). We estimated SARS-CoV-2 seroprevalence, distinguishing between antibodies against the spike (S) and nucleocapsid (N) proteins. This distinction is relevant because currently deployed vaccines elicit an immune response against the S protein, so the presence of N antibodies could be interpreted as a proxy for postinfection seroprevalence in highly vaccinated populations.

**Figure 1:**
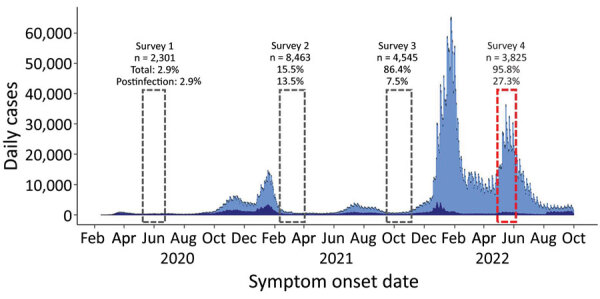
Daily number of cases and percentages of SARS-CoV-2 seroprevalence in 4 serial seroepidemiologic surveys in Portugal, May 2020–June 2022. Tick marks correspond to the first day of the month.

**Figure 2:**
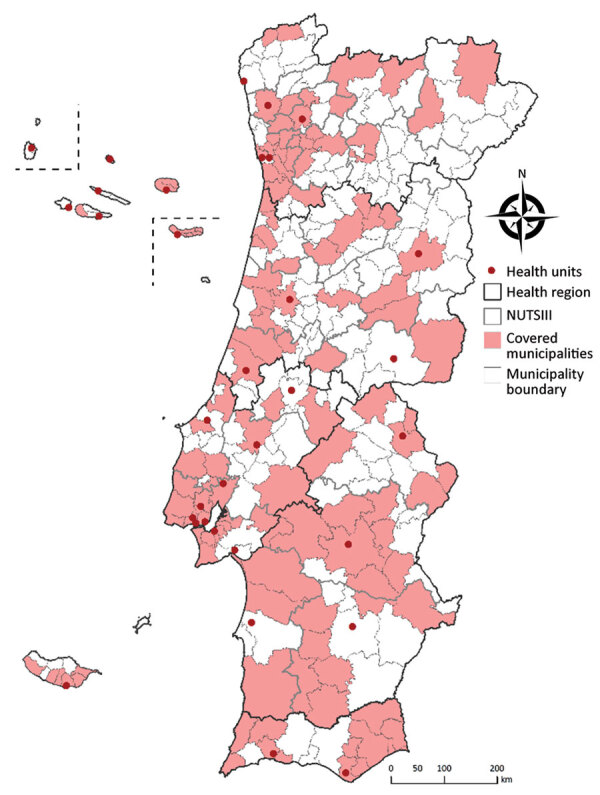
Data collection sites for study of seroprevalence of specific SARS-CoV-2 antibodies during the Omicron BA.5 wave, Portugal, April–June 2022 (ISN4COVID-19 study). NUTSIII, territorial units for statistics level III.

The study was approved by the Ethics Committee of the National Health Institute Doutor Ricardo Jorge. The need for participants’ informed consent was waived by the Ethics Committee because of the irreversible anonymization of the data at collection sites.

## The Study

Using a 2-stage nonprobability quota sampling design, we collected 3,825 irreversibly anonymized residual serum samples from persons who had undergone blood testing for reasons unrelated to COVID-19 in a nationwide network of participating clinical pathology laboratories and public hospitals (Figure 2; Appendix). For each sample, we qualitatively determined the type of IgG against SARS-CoV-2 N protein and quantitatively determined IgG against S protein by using Abbott SARS-CoV-2 Chemiluminescent Microparticle Immunoassays in the ARCHITECT i1000SR (https://www.abbott.com). We considered samples with S IgG levels >50 arbitrary units (AU)/mL to be positive. Total seroprevalence was defined as positivity for S or N IgG.

We stratified seroprevalence by patient sex, age group, and region of residence. To compare seroprevalence between population subgroups, we used a design-adjusted χ^2^ test (*7*). We described the distribution of quantitative S IgG in terms of geometric means and respective 95% CIs (Appendix). We stratified estimates by patient age group, and sex. To account for sampling design, we weighted all estimates to match the distribution of the Portugal population by sex, age group, and region of residence. We conducted statistical analyses by using Stata 15.1 software (StataCorp LLC, https://www.stata.com).

From 3,825 collected residual serum samples, 27.3% were positive for N IgG, 95.0% for S IgG, and 95.8% for either N or S IgG (Table 1). Seroprevalence of N IgG was similar by sex but varied significantly by age group, highest among children (39.2% among those 0–4 years of age and 40.0% among those 10–19 years of age) and lowest (17.3%) among adults >70 years of age. The age-related pattern for seroprevalence of S IgG differed; estimated rates were lower among those 0–4 years of age (71.2%) and 5–9 years of age (78.2%) than for those in the remaining age groups. S IgG seroprevalence also was lower in Algarve (91.0%) than in other regions of Portugal. Total seroprevalence also varied by region and age group, showing patterns similar to those of S IgG.

**Table 1:** Seroprevalence of specific SARS-CoV-2 antibodies, stratified by sex, age group, and region of residence, Portugal, April 27–June 8, 2022*

Characteristic	N IgG positive, % (95% CI)	S IgG positive, % (95% CI)	N or S IgG positive, % (95% CI)
Overall	27.3 (25.5–29.1)	95.0 (94.2–95.7)	95.8 (95.0–96.4)
Sex	p = 0.8789	p = 0.9801	p = 0.8544
M	27.4 (25.0–30.0)	95.0 (93.9–95.9)	95.7 (94.6–96.6)
F	27.1 (24.7–29.7)	95.1 (93.7–96.1)	95.8 (94.6–96.8)
Age group, y	p<0.001	p<0.001	p<0.001
0–4	39.2 (32.3–46.5)	71.2 (64.4–77.2)	76.2 (69.6–81.7)
5–9	32.9 (26.6–39.9)	78.2 (71.5–83.6)	78.7 (72.1–84.1)
10–19	40.0 (35.0–45.2)	95.2 (92.5–97.0)	96.2 (93.7–97.7)
20–29	29.7 (24.8–35.2)	97.9 (95.7–99.0)	98.6 (96.5–99.5)
30–39	30.3 (25.2–36.0)	96.3 (93.4–98.0)	96.9 (94.1–98.4)
40–49	27.2 (22.3–32.7)	96.6 (93.8–98.2)	97.7 (95.3–98.9)
50–59	27.0 (22.2–32.4)	97.5 (95.0–98.8)	97.7 (95.1–98.9)
60–69	21.0 (16.7–26.0)	97.0 (94.4–98.4)	97.4 (94.8–98.7)
>70	17.3 (13.5–21.7)	96.7 (94.2–98.2)	97.2 (94.8–98.6)
Region of residence	p = 0.1465	p = 0.0141	p = 0.0315
Norte	24.8 (21.6–28.3)	96.4 (94.9–97.5)	96.8 (95.3–97.8)
Centro	28.2 (24.3–32.6)	95.2 (93.0–96.7)	95.9 (93.8–97.3)
Lisboa e Vale do Tejo	29.3 (26.3–32.6)	94.2 (92.5–95.6)	95.3 (93.7–96.5)
Alentejo	25.4 (21.7–29.5)	95.3 (93.1–96.9)	96.0 (93.9–97.3)
Algarve	27.6 (23.2–32.5)	91.0 (87.6–93.5)	91.7 (88.4–94.1)
Madeira	31.0 (26.8–35.5)	94.1 (92.0–95.7)	95.6 (93.7–97.0)
Açores	24.4 (20.6–28.6)	93.7 (91.1–95.6)	95.9 (93.8–97.3)

*ISN4COVID-19 study. Weighted to match the distribution of the resident population of Portugal (Census 2021) by sex, age group, and region. p values indicate design-adjusted χ^2^ testing used to compare seroprevalence by sex, age group, and region. N, nucleocapsid protein; S, spike protein.

We observed lower S IgG levels among children <10 years of age (geometric mean 180.4 AU/mL for 0–4 and 426.6 AU/ml for 5–9 years of age). S IgG levels were also lower among persons >70 years of age (geometric mean 4,558.5 AU/mL) than among middle-aged adults. Higher S IgG levels were observed among those positive for N IgG (Table 2).

**Table 2:** Geometric mean of specific antibodies against SARS-CoV-2 spike protein IgG, stratified by patient sex, age group, and region of residence, Portugal, April 27–June 8, 2022*

Characteristic	Geometric mean, AU/mL (95% CI )
Sex	
M	4,344.8 (3,909.5–4,828.6)
F	4,662.3 (4,150.5–5,237.1)
Age group, y	
0–4	180.4 (118.9–273.6)
5–9	426.6 (285.4–637.7)
10–19	4,750.8 (3,795.5–5,946.5)
20–29	6,011.5 (5,050.5–7,155.4)
30–39	5,433.3 (4,342.8–6,797.6)
40–49	6,940.7 (5,645.7–8,532.9)
50–59	7,669.3 (6,297.5–9,340.0)
60–69	5,484.6 (4,420.3–6,805.2)
>70	4,558.5 (3,708.4–5,603.4)
Region of residence	
Norte	5,020.5 (4,358.0–5,783.6)
Centro	5,020.9 (4,158.3–6,062.4)
Lisboa e Vale do Tejo	3,998.1 (3,451.9–4,630.9)
Alentejo	5,038.9 (4,177.2–6,078.3)
Algarve	2,970.2 (2,294.2–3,845.3)
Madeira	5,070.6 (4,188.3–6,138.8)
Açores	4,339.1 (3,523.1–5,344.1)
Positivity for nucleocapsid IgG	
Positive	9,233.6 (8,099.1–10,527.0)
Negative	3,452.8 (3,146.7–3,788.6)

*ISN4COVID-19 study. Weighted to match the distribution of the resident population of Portugal (Census 2021) by sex, age group, and region.

## Conclusions

The fourth observational nationwide study (ISN4COVID-19) estimated that during the early Omicron BA.5 circulation period, most residents of Portugal (95.5%, 95% CI 95.0–96.4%) had specific SARS-CoV-2 antibodies resulting from infection or vaccination. Total seroprevalence increased by ≈10 percentage points compared with findings from a previous survey developed during September–November 2021 (Figure 1) (*4*). Seropositivity in Portugal during April–June 2022 was comparable to the reported seroprevalence in Scotland during May–June 2022 (95.7%) (*8*) and to that in Navarra, Spain, during May–July 2022 (S IgG 92.7%) (*9*). Seropositivity in Portugal was also in line with the high vaccination coverage achieved in Portugal (Appendix Figure 2) (*6*).

Our results reveal a steep increase in N IgG seroprevalence for all age groups between the third and fourth surveys (Appendix Figure 3) (*4*), comparable to intensive epidemic activity in Portugal during January–June 2022 (*10*). The age-related pattern of lower N IgG seroprevalence in older age groups observed in our study is in line with age-specific SARS-COV-2 notifications to the National Epidemiological Surveillance Information System in early 2022 (*10*) and similar to results from Canada (*11*) and Navarra (*9*), which reported lower postinfection seroprevalence for the older than younger age groups. 

Regarding the pediatric population, our results demonstrate high postinfection seroprevalence among children not eligible for COVID-19 vaccination. Among children 0–4 years of age, seroprevalence was >75%, higher than estimates reported for unvaccinated pediatric populations by European Union countries at the beginning of the Omicron BA.1 wave: 28.8% among children 1–4 years of age in Ireland in January 2022 (*12*); 45% among preschool children in Italy in February 2022 (*13*); and >4-fold as high as seroprevalence among children recruited in our previous seroepidemiologic study conducted during September–October 2021 in Portugal (ISN3COVID-19) (Appendix Figure 3) (*4*). The percentage of persons seropositive for N IgG in the 0–4 year age group was lower (39.2%) than for those positive for S IgG (71.2%). This finding may be associated with a previously reported shorter half-life of N IgG (*14*). The results regarding N IgG positivity should be interpreted with caution because they may reflect only the most recent infections.

Antibody levels were lower among those at the extremes of age distribution. This finding may be related to the course of the vaccination campaign and age-related immunosenescence. Since September 2021, the first booster vaccinations were rolled out by age criteria; those in the older age groups were vaccinated earlier and experienced a more pronounced wane of antibody levels (*15*) compared with middle-aged adults who were vaccinated more recently. Although starting May 15, 2022, a second booster was offered for persons >80 years of age and for institutionalized persons, our results have not yet reflected the effect of that change. The second booster recommendation was issued during collection of ISN4COVID-19 data, and at the end of the study period, only 4.4% of the population had received the second booster (*6*).

Lower antibody levels in children may be associated with postinfection immunity; antibody levels that were lower after infection than after vaccination have been reported (*14*,*15*). Furthermore, at the time of data collection, a booster was not recommended for the pediatric population, which may also explain lower antibody levels.

Among the limitations of our study, the nonrandom sampling and recruitment strategy can result in selection bias because participants seeking clinical care might differ from the general population. Also, the study might not capture reinfections, and because seroreversion occurs without recent vaccination or infection, we were unable to estimate a cumulative number of SARS-CoV-2 infections in Portugal.

In summary, almost all persons in the Portugal population have specific antibodies against SARS-CoV-2. Even among children not eligible for vaccination, ≈75% have SARS-CoV-2 antibodies. Among adults, IgG values are higher for those in age groups who received their vaccine booster more recently.

Appendix Supplemental information for study of seroprevalence of specific SARS-CoV-2 antibodies during the Omicron BA.5 wave, Portugal, April–June 2022.
