# Supplementary material for: Seroprevalence of Specific SARS-CoV-2 Antibodies during Omicron BA.5 Wave, Portugal, April–June 2022
Source: Emerg Infect Dis. 2023 Mar;29(3):590–4. doi: 10.3201/eid2903.221546 (PMC9973687; doi:10.3201/eid2903.221546)
Supplement: Appendix — Supplemental information for study of seroprevalence of specific SARS-CoV-2 antibodies during the Omicron BA.5 wave, Portugal, April–June 2022. [file 22-1546-Techapp-s1.pdf]

Article DOI: https://doi.org/10.3201/eid2903.221546

Seroprevalence of Specific SARS-CoV-2 Antibodies during Omicron BA.5 Wave, Portugal, April–June 2022

Appendix

Sample design in ISN4COVID-19

ISN4COVID-19 was developed using a two-stage stratified non-probability quota sampling design.

The survey sample was stratified by age group, and the sample size was determined in order to estimate an expected IgG anti-N seroprevalence of 25% with an absolute precision of 5% and a design effect of 1.5 for each stratum, leading to the minimum required sample size of 3897 participants at a national level.

In the first stage, the survey sample was allocated to seven regions in order to obtain estimates with the same level of precision (+/- 5%) in each one, considering the regional SARS-CoV-2 attack rates reported by the National System of Epidemiological Surveillance (SINAVE).

The seven regions of Portugal are subdivided into 25 subregions NUTS III (Nomenclature of territorial units for statistics). The NUTS classification is a hierarchical system for dividing up the territory for statistical purposes implemented in the European Union. The NUT system was established by Commission Regulation (EC) No 1059/2003. NUT III corresponds to an administrative region with a resident population ranging between 150 000-800 000 inhabitants. To ensure geographical survey coverage, we distributed the sample by subregions NUTS III, with an allocation of sampling units proportional to the population size. Within each NUT III, we randomly selected municipalities, and, within selected municipalities, the data collection points were chosen by convenience. The distribution of data collection points is shown in Figure 2.

At the second stage, in each selected data collection point (laboratory or hospital), individuals were recruited according to a pre-established age-sex quota among

those who had a medical prescription to perform blood tests for reasons unrelated to COVID-19.

Confidence intervals for geometric mean

As the distribution of IgG anti-S was right-skewed, we used a geometric mean as a summary statistic. To compute confidence intervals for the geometric mean first we created an auxiliary log-transformed variable *u= log(anti_s level)*. The arithmetic mean of the auxiliary variable *u* and its confidence interval *[L; U]* were then computed based on an approximation to a Normal distribution. In the second step, we applied inverse transformation and converted *[L; U]* back to the original scale: *[exp(L); exp(U)]*.

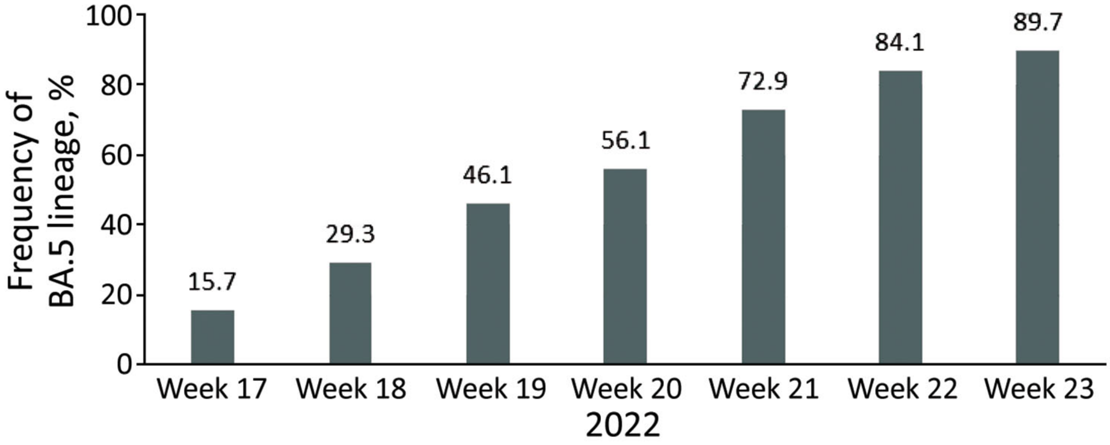

Appendix Figure 1. Evolution of the relative frequency of SARS-CoV-2 Omicron BA.5 lineage in Portugal between weeks 17 (25/05/2022-01/05/2022) and 23 (06/06/2022-12/06/2022).

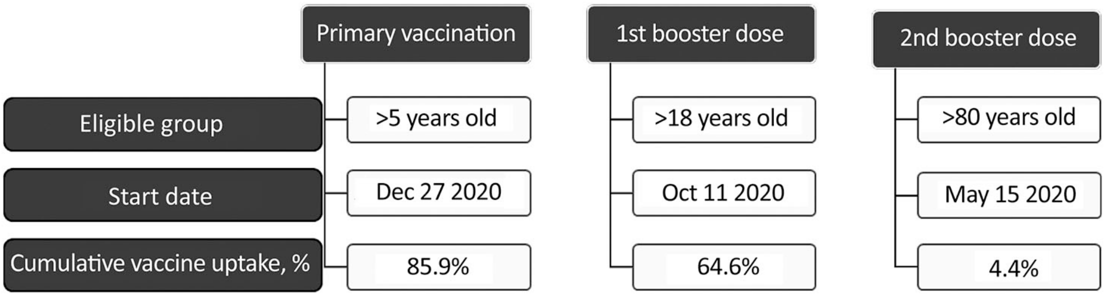

Appendix Figure 2. Cumulative uptake (%) of the primary vaccination, the first booster, and the second booster dose in the total population in Portugal, week 52, 2020 (28/12/2020-03/01/2021) - week 23, 2022 (06/06/2022-12/06/2022). Note: Cumulative vaccine uptake was obtained from the European Centre for Disease Prevention and Control COVID-19 vaccine tracker (https://vaccinetracker.ecdc.europa.eu/public/extensions/COVID-19/vaccine-tracker.html#uptake-tab). To compute cumulative vaccine uptake a total population was used as the denominator.

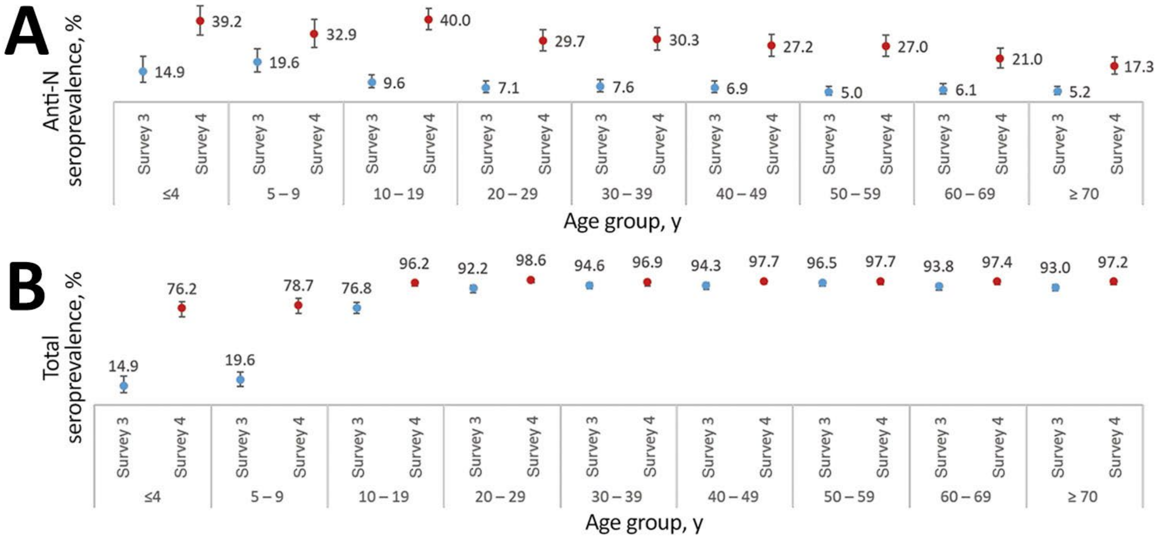

Appendix Figure 3. Evolution of SARS-CoV-2 anti-N seroprevalence (A) and total seroprevalence (B) by age group in Portugal in ISN3COVID-19 (survey3, September – November 2021) and ISN4COVID-19 (survey 4, April – June 2022). Note: Seroprevalence rates estimated by ISN3COVID-19 (survey3) are presented in blue and seroprevalence rates estimated by ISN4COVID-19 (survey4) are presented in red color.
